# Breaking the silence on suicide among pre-adolescent children

**DOI:** 10.1186/s13034-025-00999-x

**Published:** 2025-12-12

**Authors:** Moses Muwanguzi, Moses Kule, Henon Ataryeba, Godfrey Zari Rukundo, Sheila Harms, Mark Mohan Kaggwa

**Affiliations:** 1 Department of Psychiatry Faculty of Medicine, Mbarara University of Science and Technology, Mbarara, Uganda; 2 African Centre for Suicide Prevention and Research, Mbarara, Uganda; 3 Department of Psychiatry, Mbarara Regional Referral Hospital, Mbarara, Uganda; 4 Faculty of Medicine, Mbarara University of Science and Technology, Mbarara, Uganda; 5 Department of Psychiatry, University of Toronto, Toronto, Canada; 6 Waypoint Centre for Mental Health Care, Midland, Canada; 7 Department of Psychiatry and Behavioural Neurosciences, McMaster University, Hamilton, Canada

**Keywords:** Suicide, Children, Pre-adolescent, Uganda, School, Media report

## Abstract

Globally, the rising burden of suicide among pre-adolescent children is a pressing clinical problem for mental health practitioners. Over 90% of the world’s youth live in low- and middle-income countries, where suicide is the second-leading cause of death among children and youth. Literature about completed suicide in Ugandan children below 10 years of age is limited, although there is a growing body of research predominantly from high-income countries. We present the only available literature in Uganda about pre-adolescent suicide as reported in press-media reports. We utilized the multilevel risk framework to discuss the multi-sociocultural perspective regarding child rearing, the role of childhood trauma, the evolving digital environment, and legal and policy frameworks. We discussed challenges to the practice of Child and Adolescent psychiatry in Uganda, where childhood mental health disorders continue to receive limited attention in clinical practice. We recommend future research efforts to develop a robust methodology to better understand pre-adolescent suicide. Implementation of actionable interventions like school-based suicide screening, community gatekeeper trainings, and child helplines are key. Intersectoral collaborations among diverse stakeholders are essential for co-creating actionable and evidence-based preventive interventions that place the community at the centre.

## Background

Suicide is a growing international public health crisis. According to the World Health Organization (WHO), almost 1 million people die by suicide annually, which translates into one suicide death every 40 s [1]. Worldwide, rates are higher for males compared to females across all age groups, with the most considerable burden of suicides occurring in low- and middle-income countries (LMICs) [2]. Over 90% of the world’s children below 15 years of age live in LMICs, where suicide is the third-leading cause of death for 15 to 24-year-olds and the second-leading cause of death among children and adolescents aged 10–14 years [3]. Suicide among pre-adolescent children aged below 10 years has received less attention due to the long-held view that children lack the cognitive ability to comprehend death, which allegedly precludes them from deliberately engaging in suicidal behaviors [4]. However, there is a growing body of literature about pre-adolescent child suicide predominantly from high-income countries (HICs).

In North America, child suicide ranks as the eighth leading cause of death among those aged 5 to 11 years, accounting for 2.3 deaths per one million youths in 2019 [5]. These deaths are tracked through the United States (US) National Fatality Review-Case Reporting System (NFR-CRS), which reported 78 suicide incidents among children aged 6 to 9 years from 2006 to 2021 [6]. The National Violent Death Reporting System (NVDRS) on child suicide has also reported 134 suicides among children aged 5 to 11 years between 2013 and 2017 [7].

There is limited research from LMICs that explores suicide among pre-adolescent children. In India, the National Crime Record Bureau (NCRB) reported “*Accidental Deaths and Suicides in India (ADSI)*,” including 134,735 suicide deaths among children aged below 14 years between 1995 and 2021, with an increasing trend of child suicides from 2012 to 2021 [8]. Research about complete suicide among children in Africa is limited. This may be due to the absence of systematic suicide monitoring systems, compounded by stigma and innate cultural values that stigmatize suicide, resulting in underreporting [1, 2].

Descriptive characteristics of completed child suicide have found that most child suicides occurred in the child’s parental home, particularly in their bedroom, which was a finding consistently reported across both HICs and the existing LMICs’ data [7–10]. The most common methods used included hanging or suffocation, and the use of firearms that were unsafely stored in their homes [7, 9, 11]. Majority were males, mostly from black communities, who had disclosed their suicidal intent to a sibling or a friend, yet a parent was home at the time of the child’s death [7, 12].

Completed suicide among children and young people is attributed to a complex interplay between psychological, contextual, and sociocultural factors from which extreme distress originates. The majority of children reportedly experience life stressors before their death, although the exact cause remains unclear. However, studies that have used a psychological autopsy have classified possible precipitating factors for child suicide under the following four themes: [1] family-related problems [2], early childhood adversities [3], school/peer-related problems, and [4] pre-existing mental health problems [2, 4, 7, 8, 11, 12]. In addition, early access and unregulated use of smartphone technologies among children has been associated with increased risk of suicide, although evidence remains limited [13]. Although parents and guardians are typically gatekeepers to regulate social media access in homes, challenges to oversight include busy schedules as well as advancements in smart technology. The online world presents risks like cyberbullying, social comparison, new trends of imitation, and online challenges, which not only contribute to psychological distress but also expose children to suicidal content, which may further influence suicidal behavior.

### Child suicide in the Ugandan context

In Uganda, as in other LMICs, suicide among pre-adolescent children has received little attention, thus literature remains scarce. In addition to being stigmatized, viewed as culturally inappropriate, and unexpected among young children, suicide attempts remains a criminal offense in Uganda, with a sentence of a jail term of six months according to Ugandan law (the Penal Code 1950) [14]. The few studies in Uganda that explored completed suicides included children as a subsample but did not provide details about them. For example, a recent review of medical records in southwestern Uganda included pre-adolescent children (ages ranged from 8 to 98 years), with no specific details about them [15]. The majority of suicide studies in Uganda have been conducted among vulnerable groups of adolescents and young adults, with no study among pre-adolescent children. The best proxy information that is available for this under-represented group are studies that report suicidal ideation in children or family histories of suicide. Among a sample of 271 children and adolescents aged 6–18 years living with HIV/AIDS in Uganda, 17% had active suicidal ideation, of which 47.8% were aged below 13 years [16]. In another study focusing on adolescents living with HIV, a 10.7% prevalence of suicidality (suicidal ideation and/or suicidal attempt) was reported in the past month [17].

### The existing Ugandan data on child suicide

Uganda has no suicide database, and previous studies have used press media reports to understand suicide phenomenology in Uganda [18–20]. Similarly, we adopted a similar methodology by conducting an exhaustive systematic online search of Ugandan press media reports of complete suicides among pre-adolescent children between 2015 and 2024 [21–27]. These were accounts of residents, parents, and the siblings of the deceased. A team of experienced reviewers extracted information on; (i) date of suicide, (ii) age and sex, (iii) incident country region, (iv) method of suicide, and (v) circumstances preceding the suicide. Following the removal of duplicates and triangulation through multimedia source corroboration of similar incidents, a total of 5 pre-adolescent suicides were recorded [21–27], and brief descriptions are given below.

On January 12, 2019, an 8-year-old girl from Northern Uganda was reportedly reprimanded by her aunt for getting her clothes dirty while playing with friends. She reportedly died by hanging after confiding in her young sister about her suicidal plan [22]. On June 12, 2020, a 7-year-old boy from Central Uganda had reportedly been instructed by his mother to mop their house shortly after lunch, but disappeared and was found hanging in his bedroom [23]. On January 12, 2022, a 6-year-old girl residing in Central Uganda reportedly completed suicide by hanging, protesting her transfer back to school in her local village, following Uganda’s post–COVID-19 school reopening [24]. On June 21, 2022, a 9-year-old girl from Central Uganda reportedly stole charcoal from neighbors to prepare tea for the family. She hanged herself after her theft was discovered and was about to be reported to her mother [25, 27]. A 9-year-old boy in southwestern Uganda completed suicide on November 24, 2023, by hanging after being detained alone in the school dormitory due to the failure of his parents to pay outstanding school fees [26, 27]. The above press media reports present anecdotal evidence that has not been systematically validated. Interpretation may be limited due to bias from press media sensational reporting of the incidents to attract readership, which may distort and overshadow the nuanced realities of each pre-adolescent suicide. In addition, we may not have captured incidents that happen in hospitals following suicide attempts and those not reported in communities owing to the criminalization and stigmatization of suicide.

Methodological limitations aside, future research is urgently warranted to uncover the complex pre-adolescent vulnerabilities while exploring context-specific multicultural community-driven co-created sustainable interventions. All stakeholders in child mental health should focus on this missed opportunity to prevent such premature deaths, and the discussion below highlights some of the risk factors and recommended ways forward from a multicultural biopsychosocial perspective, utilizing the multilevel risk model [28].

## Discussion

Although suicide is a highly prevalent global mental health problem, it is generally unexpected among pre-adolescent children. However, literature from HICs shows that pre-adolescent suicide is a common cause of death, and trends are tracked by the national suicide databases. We discuss potential risk factors for pre-adolescent suicide, following the multilevel risk framework. This is an integrative framework that demonstrates the interplay and interdependency of risk factors nested within multiple levels to understand how these layers influence the occurrence of pre-adolescent suicides [28]. This approach accommodates a multi-sociocultural perspective, the role of childhood trauma, psychiatric practice, and digital environment influences in a structured way to clarify their complex interplay.

### Multi-sociocultural perspective

Mental health of children is complexly determined by their sociocultural environment, which extends from their home to being a community role. Negative cultural beliefs about suicide in Africa have contributed to widespread suicide illiteracy, limiting open discussion and preventive action. In addition, the criminalization of suicidal behaviors in many African countries (like Uganda) has reinforced the negative cultural beliefs about pre-adolescent suicide. This has made research and policy discussions on pre-adolescent suicidal behaviors difficult, thus creating a mental health gap critical to be addressed through multi-stakeholder engagements.

### Role of childhood trauma

There is emerging evidence that over half of child and adolescent mental disorders (including suicide) are a result of exposure to adverse childhood experiences (ACEs) (like child abuse, neglect, household dysfunctions) [29, 30]. Increasing exposure to intimate partner violence, armed conflicts, unstable political environments, food insecurities, child labour, forced migrations, orphanhood, and poverty, among others, may ultimately affect resilience, coping mechanisms to stressful environments resulting into suicidal behaviors [31].

Polyvictimization of children has been reported to increase the odds of impulsive suicidal behavior among young people, although such studies have not been conducted among pre-adolescent children [32]. Therefore, efforts for suicide prevention should address childhood trauma through building awareness to create more culturally sensitive and child-friendly communities for our children.

### Practice of child and adolescent psychiatry

There is a widening child and adolescent mental health gap, especially due to demographic transitions and the scarcity of mental health personnel dedicated to screening, diagnosing, and managing affected children and their families [33]. In addition, a lack of culturally sensitive screening tools, community awareness, and stigma contribute to late diagnosis, which complicates management [33]. Therefore, undiagnosed or poorly managed child mental illness, like depression, attention-deficit/hyperactivity disorder (ADHD), could increase the risk for impulsive suicidal behaviors [34]. More efforts are needed to build capacity to improve the contemporary practice of child and adolescent psychiatry to improve the quality of individualized care.

### Role of the evolving digital environment

Research has demonstrated that youth can be traumatized through exposure to virtual suicidal content on social media platforms [35]. The United Nations International Children’s Emergency Fund (UNICEF) reports that over 80% of children in 25 countries report feeling in danger of sexual abuse or exploitation online [36]. UNICEF works to make the internet a safe place for children to learn, socialize, and express themselves through projects like the *Global Kids Online* and *Disrupting Harm* projects to gather evidence on children’s digital rights, opportunities, and risks to better understand how the use of digital technology contributes to their lives, and when it amplifies their risk of harm [37]. Suicide prevention efforts should also involve the regulation of the content to which children are exposed through child safety subscriptions and parental guidance options on the devices.

### Legal and policy framework on suicide

Current policies on suicide in Uganda pose significant threats to addressing suicide as a public health mental health problem, which highlights significant gaps that counter access to care and to suicide surveillance [33]. Despite rising suicide rates, Uganda lacks a comprehensive national suicide prevention strategy integrated into its mental health policy, limiting coordinated responses to childhood and adolescent mental health needs [33]. Policies on child protection and mental health access remain insufficiently enforced, with stigma surrounding suicide and mental illness further discouraging help-seeking behaviors [38]. Resource allocation for mental health services is grossly insufficient, especially in rural and underserved regions, compounding barriers to timely intervention [39]. Additionally, policies criminalizing attempted suicide foster stigma and inhibit the development of supportive environments, worsening psychosocial stressors that elevate suicide risk in children and adolescents [39]. Together, these fragmented legal and policy factors create a socio-legal environment that inadvertently sustains vulnerability and impedes effective prevention efforts in Uganda.

### Recommendations to relevant stakeholders

The lack of a suicide database greatly limits suicide research in low-income countries, since stakeholders have limited evidence base to guide the development and implementation of interventions. A comprehensive suicide monitoring system should be established and integrated into existing health monitoring systems, like incorporating suicidality assessment modules into Demographic and Health Surveys, and routine hospital surveillance systems. This will enhance suicide monitoring and will provide real-time data to guide clinical practice and inform policy reforms.

Interventions to increase suicide literacy among pre-adolescent children should be multi-pronged and targeted at all stakeholders involved in child and adolescent health. For example, school-based mental health screening programs can identify at-risk children early and linkage to mental health professionals can be done timely. Additionally, community gatekeeper training can equip teachers, parents, and community leaders to recognize warning signs of a distressed child and respond appropriately. Child-focused helplines and digital platforms may improve access to culturally sensitive support for children in distress. Efforts should also include programs to enhance suicide literacy among pre-adolescents, caregivers, and community members, addressing cultural stigma and policy barriers that hinder help-seeking.

Such initiatives require strong intersectoral collaboration across health, education, and Information Communication Technology (ICT), and regulation sectors to ensure coordinated, sustainable, and contextually relevant interventions for children’s mental health, while addressing known risk factors. Such a collaborative approach will enhance data collection, improve early detection, and strengthen preventive strategies, ultimately reducing suicide risk among pre-adolescents.

## Conclusion

In conclusion, pre-adolescent suicide devastates the family and the larger community due to its long-lasting psychological and social impacts of the loss of a young life. Child suicide constitutes a sociocultural phenomenon that requires further exploration. Research should address the urgent need for understanding risk and protective factors for suicidal ideation, planning, and attempt among pre-adolescent children to halt progression to completion of suicide. Suicide monitoring systems are key in tracking the evolving trends and demographic influences of suicide to generate evidence to support future interventions.

## Data Availability

No datasets were generated or analysed during the current study.

## Abbreviations

UNICEF: United Nations International Children’s Emergency Fund
ACEs: Adverse childhood experiences
NCRB: National Crime Record Bureau
ADSI: Accidental Deaths and Suicides in India
NVDRS: National Violent Death Reporting System
NFR-CRS: National Fatality Review-Case Reporting System
HICs: High income countries
LMICs: Low- and middle-income countries
WHO: World Health Organization
